# Nicotinamide-mediated inhibition of SIRT1 deacetylase is associated with the viability of cancer cells exposed to antitumor agents and apoptosis

**DOI:** 10.3892/ol.2013.1400

**Published:** 2013-06-14

**Authors:** TONG WANG, HUIXIA CUI, NAN MA, YOUHONG JIANG

**Affiliations:** 1 Department of Medical Oncology, China Medical University, Shenyang, Liaoning 11001;; 2 College of Nursing, Liaoning Medical University, Jinzhou, Liaoning 121001, P.R. China

**Keywords:** SIRT1, deacetylase, stress response, nicotinamide, apoptosis

## Abstract

Silent mating-type information regulation 2, homolog 1 (SIRT1) represents an NAD^+^-dependent deacetylase that regulates the processes of stress response and cell survival. However, the functions of SIRT1 in stress- and drug-induced apoptosis remain elusive. The present study was designed to determine the effects of SIRT1 in tumor cells subjected to antitumor agent treatment and to identify the underlying mechanisms during the stress response. Several of the most commonly used antitumor medications [arsenic trioxide (As_2_O_3_), Taxol and doxorubicin (doxo)] were selected to treat MCF-7 human breast cancer cells with or without nicotinamide (NAM) inhibition. 3-(4,5-Dimethyl-2-thiazolyl)-2,5-diphenyl-2H-tetrazolium bromide (MTT) was used to test cell viability. SIRT1 expression was tested by immunoblot analysis. The typical hallmarks of apoptosis (chromatin condensation, apoptotic bodies, sub G_1_ change and Annexin V^+^/PI^−^ stained cells) were detected by Hoechst 33342 staining, flow cytometry and Annexin V^+^/PI^−^ staining following NAM treatment. The cleavage of poly(ADP-ribose) polymerase (PARP) and caspases 9, 6 and 7 was detected through immunoblot analysis. Augmented SIRT1 expression was observed only at low concentrations (>80% cell viability) and the inhibition of SIRT1 deacetylase by NAM decreased the viability of the cancer cells exposed to low concentrations of antitumor agents. NAM induced typical apoptosis in the MCF-7 tumor cells, accompanied by the activation of the caspase cascade. SIRT1 promotes cellular survival at certain stress levels by its deacetylase function. The SIRT1 deacetylase inhibitor, NAM, triggers the activation of the caspase cascade and induces typical apoptosis in MCF-7 cells.

## Introduction

The sirtuins, or SIRTs, are highly conserved mammalian homologues of yeast silent mating-type information regulation 2, homolog (Sir2), which catalyze NAD^+^-dependent histone deacetylation and ADP ribosylation (1). Numerous studies have shown that the levels of silent mating-type information regulation 2, homolog 1 (SIRT1) are significantly elevated in prostate, ovarian, gastric and colorectal cancer, as well as hepatocellular carcinoma (2–6). Moreover, SIRT1 inhibition has been reported to suppress cell growth and induce cell cycle arrest or apoptosis in cancer cells (7). Although SIRT1 has emerged as a key regulator in various cellular pathways, the regulatory mechanisms responsible for SIRT1 activity have not been determined. SIRT1, the mammalian homolog of Sir2, has been shown to regulate a wide variety of cellular processes (8,9), including glucose metabolism (10,11), the cell cycle, growth and differentiation, inflammation, senescence, apoptosis (12), the stress response (13) and aging.

The present study focused on the role of SIRT1 in the stress response. Previous studies have shown that Sir2 represses p53-dependent apoptosis in response to DNA damage and oxidative stress by physically interacting with p53 (13) and the forkhead transcription factor (FOXO) family of proteins (14), indicating that SIRT1 promotes cellular survival. However, embryonic stem cells and fibroblasts from SIRT1-null mice showed no altered resistance to DNA damage-induced stress (15). In tumor cells, SIRT1 also failed to alter cell survival following DNA damage (16).

The controversial functions of SIRT1 require investigation as to i) whether SIRT1 has biological function in tumor cells subjected to antitumor agent treatment; ii) how SIRT1 executes its function during the stress response; and iii) what would happen to tumor cells if the deacetylase activity of SIRT1 was inhibited. For the present study, nicotinamide (NAM), the most potent inhibitor of Sir2 enzymes to date (17–20), was used to inhibit the deacetylase activity of SIRT1.

## Materials and methods

### Cell culture and reagents

Human breast cancer MCF-7 cells were seeded at 1×10^5^ cells/well (n=2 for each condition) in 24-well tissue-culture plates containing 0.5 ml complete medium (RPMI-1640 medium supplemented with 10% fetal bovine serum; Tianjin Haoyang Biotech Company, Tianjin, China) and 2 mM glutamine, penicillin and streptomycin (100 units/ml). The cells were incubated at 37°C in a humidified atmosphere with 5% CO_2_. The study was approved by the ethics committee of China Medical University (Shenyang, P.R. China).

NAM was prepared as a 1 M solution with phosphate-buffered saline (PBS) and stored at −20°C ready for use. Doxorubicin (doxo), arsenic trioxide (As_2_O_3_) and Taxol were purchased from Sigma-Aldrich Chemical Inc. (St. Louis, MO, USA). 3-(4,5-Dimethyl-2-thiazolyl)-2,5-diphenyl-2H-tetrazo lium bromide (MTT), Hoechst 33342, RNase A, RPMI-1640 medium and DMEM were purchased from Invitrogen (Carlsbad, CA, USA).

### Cell viability assay

The MTT assay was used to assess cell viability. The cells were seeded into 96-well plates (4,000 cells/well) for 24 h and treated with various anti-tumor agents for 72 h. Subsequently, 20 *μ*l MTT (5 mg/ml) was added to the medium. Subsequent to incubation at 37°C for 4 h, the culture medium containing MTT was removed and 200 *μ*l DMSO was added to solubilize the blue formazan formed by the viable cells. The plates were read with an ELISA plate reader at 570 nm. Cell viability is presented as a percentage ratio of exposed cells to control cells.

### Immunoblot analysis

The treated cells were scraped from the culture, washed with PBS and lysed with buffer containing 25 mM Tris-HCl (pH 7.5) 150 mM NaCl, 2 mM EDTA, 10% glycerol, 10 mM glycerophosphate, 5 mM sodium pyrophosphate, 5 mM NaF, 1 mM Na_3_VO_4_, 0.5% Triton X-100, 1 mM PMSF, 2 *μ*g/ml aprotinin and 2 *μ*g/ml leupeptin. Equal amounts of protein samples were loaded onto SDS-PAGE gels and transferred to PVDF membranes, then probed with the corresponding antibodies. Antibodies directed against poly(ADP-ribose) polymerase (PARP), cleaved caspase 6, cleaved caspase 7 and cleaved caspase 9 were obtained from Cell Signaling Technology (Beverly, MA, USA), while anti-SIRT1 antibody was purchased from Santa Cruz Biotechnology Inc. (Santa Cruz, CA, USA). The protein signals were detected with an enhanced chemiluminescence system (RPN 2106) according to the manufacturer’s instructions (Amersham Biosciences, Indianapolis, IN, USA).

### Detection of chromatin condensation with Hoechst 33342 staining

The cells (2×10^5^ per well) were seeded into a six-well plate for 24 h and treated with NAM for the indicated times. The suspended and adherent cells were collected and incubated with 2 *μ*g/ml Hoechst 33342 at 37°C for 30 min. Chromatin condensation was observed by fluorescence microscopy.

### Cell cycle analysis

The treated cells were trypsinized and washed once with PBS, then fixed with cold 75% ethanol overnight. The fixed cells were washed twice with PBS and incubated with 100 *μ*g/ml RNase I and 50 mg/ml propidium iodide (PI) for 30 min, then the cellular DNA content was determined by flow cytometry (FACS Calibur, BD Biosciences, Franklin Lakes, NJ, USA).

### Assessment of apoptosis by Annexin V^+^/*PI*^−^ staining

The apoptotic cells were detected by the Apop Nexin^TM^ FITC Apoptosis Detection kit (Chemicon, Temecula, CA, USA) according to the manufacturer’s instructions. Briefly, the suspended and adherent cells were pooled, washed twice with ice-cold PBS and resuspended in binding buffer to 10^6^/ml. Next, 0.2 ml of this cell suspension was incubated with 3 *μ*l fluorescein isothiocyanate (FITC)-labeled Annexin V and 2 *μ*l PI for 60 min at room temperature in the dark. Samples were analyzed by flow cytometry.

### Statistical analysis

Each experiment was repeated at least three times. Data are presented as the mean ± standard deviation.

The combined effects of the antitumor agents and NAM on cell viability were calculated according to the coefficient of drug interaction (CDI) (21), calculated by the formula: CDI = AB / (A × B), where AB represents the cell viability of the combination of drug A and B, while A or B represent cell viability of the single compound alone. CDI <1, CDI = 1 and CDI >1 represent the synergy, additivity and antagonism of A and B, respectively.

## Results

### SIRT1 expression increases under certain stress response levels

In order to study the biological functions of SIRT1 in the stress response caused by antitumor agent treatment, As_2_O_3_, Taxol and doxo, the most commonly used clinical antitumor agents, were selected according to their different mechanisms of action. As_2_O_3_ is an effective therapy in acute promyelocytic leukemia (APL) (22) and has also exhibited promising activities in other hematological and solid tumors (23,24). As_2_O_3_ targets differentiation, apoptosis and protein oxidative damage (25). Taxol binds microtubules and causes the kinetic suppression of microtubule dynamics, thus killing cancer cells through the induction of apoptosis (26,27). Topoisomerase II is generally recognized to be the main cellular target of doxo. There appears to be general agreement that oxidative stress is a significant contributor to the antitumor activity of doxo (28,29).

**Figure 1.**
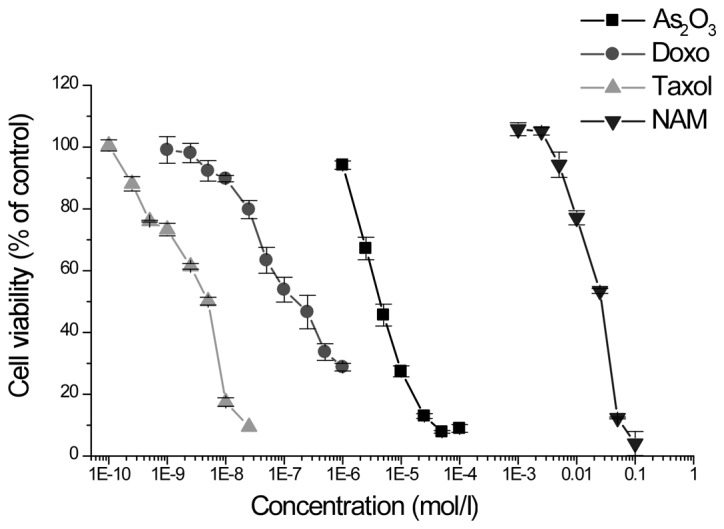
Effects of doxo, Taxol, As_2_O_3_ and NAM on MCF-7 cell viability. Various concentrations led to dose-dependent inhibition of cell proliferation, as detected by MTT assay. Doxo, doxorubicin; NAM, nicotinamide; As_2_O_3_, arsenic trioxide; MTT, 3-(4,5-dimethyl-2-thiazolyl)-2,5-diphenyl-2H-tetrazolium bromide.

First, the anti-proliferative effects of doxo, Taxol, As_2_O_3_ and NAM were examined with the MTT assay. Incubation of MCF-7 cells with various concentrations of doxo, Taxol, As_2_O_3_ and NAM led to the dose-dependent inhibition of cell proliferation (Fig. 1). The IC_50_ values obtained subsequent to 72 h of treatment were (1.7±0.1)×10^−7^, (2.9±0.2)×10^−9^, (5.4±0.4)×10^−6^ and (21.5±0.7)×10^−3^ mol/l for doxo, Taxol, As_2_O_3_ and NAM, respectively.

**Figure 2.**
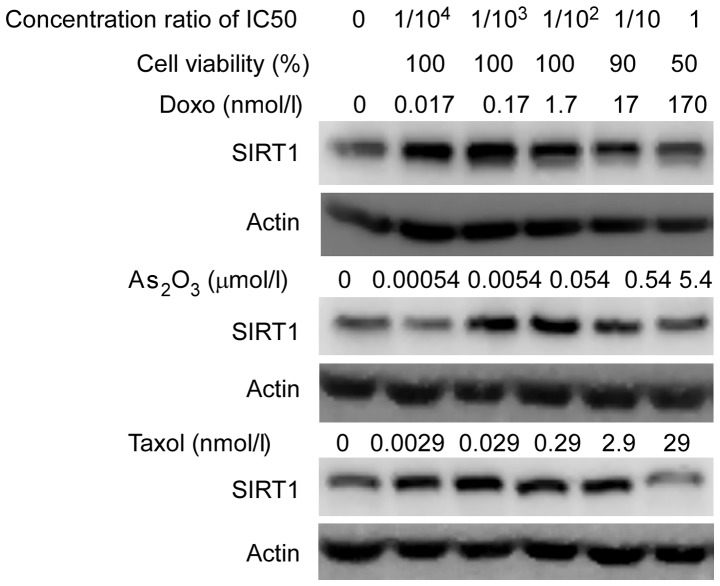
SIRT1 expression in MCF-7 cells treated with the antitumor agents doxo, As_2_O_3_ and Taxol at various concentrations for 24 h, as detected by immunoblot analysis. SIRT1, mammalian homolog of Sir2; doxo, doxorubicin; As_2_O_3_, arsenic trioxide.

Subsequently, the MCF-7 cells were treated with doxo, Taxol and As_2_O_3_ at various concentrations, which led to 100%, 90% and 50% cell viability, respectively, and SIRT1 expression was detected by immunoblot analysis following antitumor agent treatment for 24 h. Notably, increased expression levels of SIRT1 were observed at low concentrations (>90% cell viability), but not at high drug concentrations (50% cell viability; Fig. 2). This result may suggest that only the stress levels that lightly damage tumor cells are able to activate the SIRT1 pathway.

### NAM decreases the viability of MCF-7 cells through SIRT1 deacetylases

**Figure 3.**
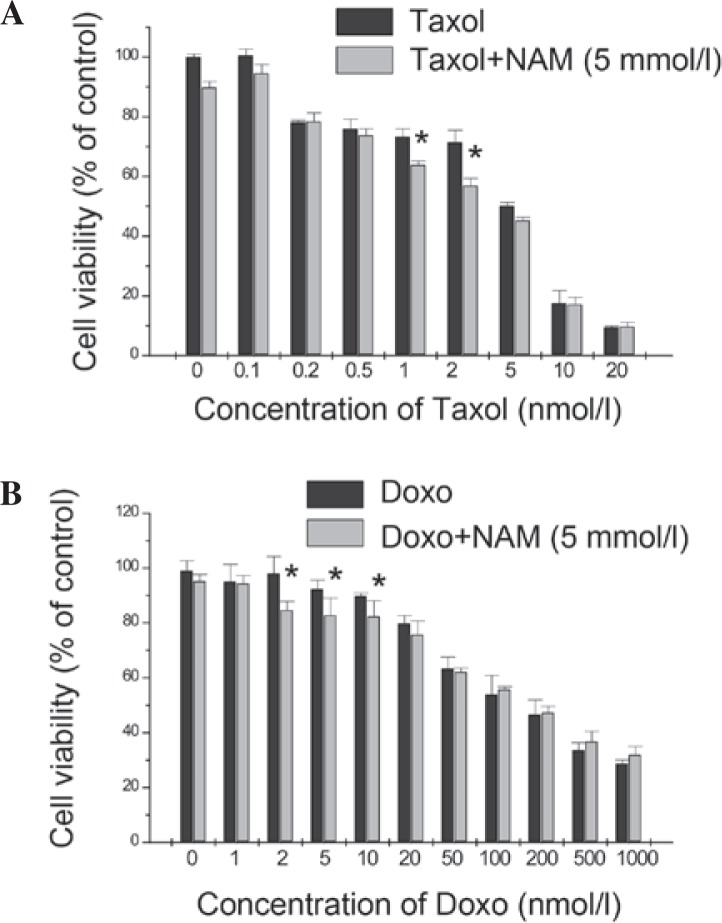
NAM decreased the viability of MCF-7 cells, which were exposed to (A) Taxol and (B) doxo via a synergistic effect, as detected by MTT assay. NAM decreased the viability of MCF-7 cells at low drug concentrations in each case. Error bars indicate deviations from three independent experiments performed in triplicate. ^*^CDI<1. NAM, nicotinamide; doxo, doxorubicin; MTT, 3-(4,5-dimethyl-2-thiazolyl)-2,5-diphenyl-2H-tetrazolium bromide; CDI, coefficient of drug interaction.

It is well known that SIRT1 is a nuclear enzyme with deacetylase activity, and the present study aimed to investigate what happened if SIRT1 deacetylates were inhibited during the stress response. Therefore, NAM was added during antitumor agent treatment to inhibit SIRT1 deacetylase, and the change in cell viability was detected with the MTT assay. NAM at 5 mM was selected to inhibit SIRT1 deacetylase since it is non-toxic and remains active at this concentration (17–20). It is worth noting that NAM decreased the viability of the MCF-7 cells exposed to doxo and Taxol at low drug concentrations and that NAM had a synergistic effect only with 1 and 2.5 nmol/l Taxol (CDI, 0.96 and 0.88, respectively; Fig. 3A), while a similar result was obtained with the combined usage of doxo and NAM (CDI<1; Fig. 3B). This result indicates that SIRT1 promotes cancer cell survival under certain conditions of applied cellular stress and that this activity is mediated by its deacetylase activity.

### Apoptosis is induced by NAM in MCF-7 cells

**Figure 4.**
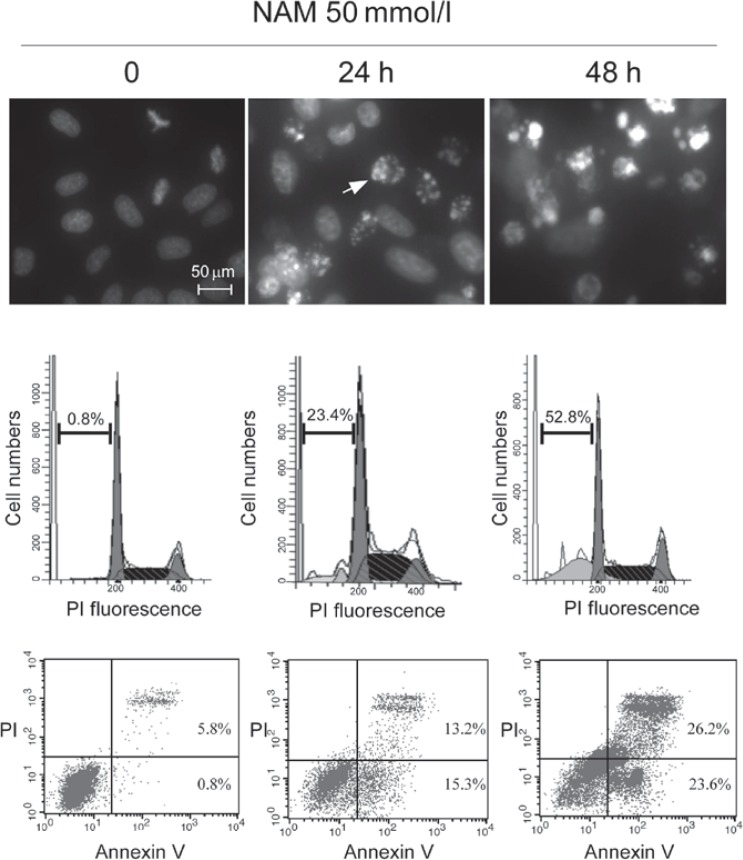
Indications of NAM-induced apoptosis in MCF-7 cells (all occurred in a time-dependent manner). (A) Chromatin condensation and apoptotic bodies (as indicated by white arrow), detected by Hoechst 33342 staining. (B) Increased sub-G_1_ cells population, detected by flow cytometry. (C) Increased Annexin V protein, detected by Annexin V^+^/PI^−^ staining. NAM, nicotinamide; PI, propidium iodide.

The subsequent aim was to investigate whether the inhibition of the deacetylase activity of SIRT1 with NAM was able to induce apoptosis. MCF-7 cells were exposed to 50 mmol/l NAM for 24 and 48 h, and typical biochemical hallmarks of apoptosis (30), such as chromatin condensation (Fig. 4A), sub G_1_ cell cycle distribution and Annexin V^+^/PI^−^ stained cells, were detected to demonstrate the occurrence of apoptosis. In Fig. 4A, a considerable amount of chromatin condensation and apoptotic bodies (as indicated by the white arrow) were observed in the NAM-treated cells assessed by fluorescence microscopy. For the flow cytometry, an increased sub-G_1_ cell cycle population (Fig. 4B) and a greater number of Annexin V^+^/PI^−^ staining cells (Fig. 4C) were detected in the NAM-treated cells. These data indicated that NAM induces typical apoptotic features in MCF-7 cells.

**Figure 5.**
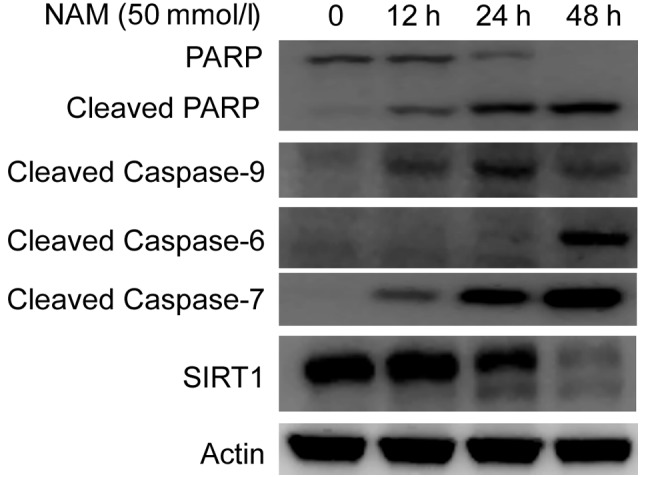
Activation of the caspase cascade in NAM-induced apoptosis in MCF-7 cells, as detected by immunoblot analysis. Cleaved PARP and caspases, as well as SIRT1 degradation, occurred in a time-dependent manner. NAM, nicotinamide; PARP, poly(ADP-ribose) polymerase; SIRT1, silent mating-type information regulation 2, homolog 1.

Apoptosis is a tightly controlled multi-step process of cell death, with the orderly involvement of proteins, such as initiator caspase 9 and executioner caspases 6 and 7, which then cleave PARP when activated. To determine the role of the caspase cascade in NAM-induced apoptosis, the MCF-7 cells were exposed to 50 mmol/l NAM for 12, 24 and 48 h, respectively. The activation of caspases and PARP was detected by cleavage fragments in the immunoblot analysis. The results (Fig. 5) showed that NAM triggered the activation of caspases 9, 6 and 7 and the cleavage of PARP in a time-dependent manner. Together, this led to the conclusion that NAM induces apoptosis in tumor cells, accompanied by the activation of the caspase cascade.

## Discussion

In the present study, SIRT1 showed an increased expression with low concentrations of drug treatment, but no altered expression at high concentrations. We propose that different drug concentrations may cause different degrees of cellular damage, which arouse various biological effects (31,32), and also that the SIRT1 pathway was activated only at the early phase of drug treatment at sub-lethal concentrations. It has been reported that biopsies from cancer patients treated with chemotherapeutic agents also expressed high levels of SIRT1 (33). Consequently, SIRT1 may be regarded as a potential target for the diagnosis and treatment of cancer chemotherapy. It was also noted that the inhibition of SIRT1 with NAM sensitized the MCF-7 cells to drug treatment only at low concentrations, demonstrating that the activated SIRT1 pathway promoted tumor cell survival through its deacetylase activity. This conclusion leads to a novel means of improving the clinical therapeutic effect of tumor chemotherapy.

SIRT1 is an enzyme that catalyzes the deacetylation of acetyl-lysine residues by a mechanism in which NAD^+^ is cleaved and O-acetyl ADP-ribose is generated. The reaction results in the release of NAM, a form of Vitamin B3 that acts as an end product inhibitor (15). NAM has been shown to increase radiosensitivity in the course of cancer radiotherapy. NAM is considered to reduce the occurrence of acute hypoxia and hence increase tumor blood flow (34,35), although the precise mechanism of action remains unclear. In the present study, NAM was used to examine the role of SIRT1 in the stress response and it was observed that NAM had a synergistic effect with low concentrations of antitumor agents, thus increasing chemosensitivity in the course of cancer chemotherapy. The results also suggested that increased radiosensitivity by NAM may occur via SIRT1 inhibition.

It has been reported that silencing SIRT1 gene expression by RNA interference (RNAi) induces growth arrest and apoptosis in human epithelial cancer cells. By contrast, normal human epithelial cells and normal human diploid fibroblasts appear to be refractory to SIRT1 silencing. Therefore, SIRT1 may be identified as a novel target for the selective killing of cancer instead of non-cancer epithelial cells (36). In the present study, the SIRT1 deacetylase inhibitor, NAM, induced typical apoptotic features in the MCF-7 tumor cells. Concentration of 50 mM NAM may be too high for clinical application, although more sensitive SIRT1 inhibitors, such as indole and EX527, have been identified as potent and selective inhibitors of the deacetylase SIRT1 (37).
